# Supplementary material for: Radioiodine in Differentiated Thyroid Carcinoma: Do We Need Diagnostic Pre-Ablation Iodine-123 Scintigraphy to Optimize Treatment?
Source: Diagnostics (Basel). 2021 Mar 19;11(3):553. doi: 10.3390/diagnostics11030553 (PMC8003652; doi:10.3390/diagnostics11030553)
Supplement: Supplementary file 1 [file diagnostics-11-00553-s001.zip › diagnostics-1148010-supplementary (1)/Table S2.docx]

**S2 Table. Unsuccessful treatment after nine months.** N=56.

|  | *Suspected persistent disease (n=22)* | *Persistent / progressive disease (n=34)* |
| --- | --- | --- |
| ***Biochemical evidence of disease*** |  |  |
| sTg 0.5-1.0 | 4 (18%) | 3 (9%) |
| sTg >1.0 | 0 (0%) | 26 (76%) |
| Positive anti-Tg antibodies | 6 (27%) | 5 (15%) |
| ***Structural evidence of disease*** |  |  |
| Suspicious US of thyroid bed | 7 (32%) | 7 (21%) |
| Persistent uptake in thyroid bed on DxWBS | 2 (10%) | 9 (26%) |
| New uptake in thyroid bed | 0 (0%) | 1 (3%) |
| Suspected lymph node metastasis on US | 7 (32%) | 11 (32%) |
| Persistent lymph node metastasis on DxWBS | 0 (0%) | 6 (18%) |
| New lymph node metastasis on DxWBS | 0 (0%) | 5 (15%) |
| Persistent distant metastasis | 0 (0%) | 6 (18%) |
| Suspected new distant metastasis on DxWBS | 1 (5%) | 0 (0%) |
