# Supplementary material for: Radioiodine in Differentiated Thyroid Carcinoma: Do We Need Diagnostic Pre-Ablation Iodine-123 Scintigraphy to Optimize Treatment?
Source: Diagnostics (Basel). 2021 Mar 19;11(3):553. doi: 10.3390/diagnostics11030553 (PMC8003652; doi:10.3390/diagnostics11030553)
Supplement: Supplementary file 1 [file diagnostics-11-00553-s001.zip › diagnostics-1148010-supplementary (1)/File S1 Supplementary material.docx]

**File S1: Supplementary Material**

**Preparation for I-131 administration**

- Thyroid hormone withdrawal until serum TSH ≥ 30mEh/L:
  - Indications: nearly all patients, with the exception of those mentioned below;
  - At least 4 weeks after surgery.
- Recombinant TSH (rhTSH):
  - Indications: low-risk thyroid carcinoma, or in case withdrawal was considered too hazardous to the patient;
  - administered 48 and 24 hours before I-131 administration.

**Table A. Dosimetry for I-131 ablative therapy.**

| **Histopathology (TNM classification)** | **I-131 Dose** |
| --- | --- |
| Classical (FV)PTC, pT1aR0 cN0 cM0/x  No or minimal thyroid remnant | none |
| Minimally invasive FTC / unifocal FTC / classical (FV)PTC, pT1a-pT2 pR0  cN0-pN1a without extranodal growth, cM0/x  No large thyroid remnant | 1.1 GBq |
| DTC with no expected remaining disease*:   - Multifocal tumour - non-classical FTC: oncocytic (Hürthle cell) - non-classical PTC: tall cell, columnar, cribriform or diffuse sclerosing type - pT3 with radical resection (R0) - pN1a with extranodal growth or limited pN1b | 3.7 GBq |
| DTC with high risk of remaining disease*:   - pT4 - microscopic irradical resection (R1) - N1b | 5.55 GBq |
| DTC with certain remaining disease*:   - macroscopic irradical resection (R2) - pN1a/b *in situ* - p/cM1 | 7.4 GBq |

* presence of any of the following.

PTC: papillary thyroid carcinoma. (FV)PTC: follicular variant of PTC. FTC: follicular thyroid carcinoma

**Thyroglobulin measurement**

During the course of the current study, at our institution, Tg was measured using the two-step immunoradiometric assay (IRMA) Tg-plus RIA (Millipore, Henningsdorf, Germany), having its assay detection limit (functional sensitivity) at 0.5 ng/ml. Serum thyroglobulin (Tg) was determined on the day of TRA or the 5^th^ day after first rhTSH administration. In this study any Tg determined at TSH levels higher or equal to 30 mEh/L, either by THW or after rhTSH administration, is referred to as stimulated Tg (sTg).

Anti-Tg antibodies (Tg-ab) are present in approximately 20% of the patients with DTC, two times higher than in the general population [1]. Depending on the laboratory method of measurement of Tg, Tg-ab interfere with the value of Tg. The IRMA method-reports falsely low levels of Tg, underestimating the disease [1-3]. Until July 2013, our institutional laboratory used a DiaSorin RIA (DiaSorin, Saluggia VC, Italy) for the measurement of Tg-ab and later using the Siemens Immulite 2000 XPi (Siemens, Erlangen, Germany). These two methods handle different scales for the amount of present Tg-ab, though their cut-off value for detectability of Tg-ab was the same. So, this change in assay did not influence interpretation of presence or absence of Tg-ab or the interpretation of Tg values.

**TxWBS qualitative assessment**

A standardized form in Dutch was used for the independent and blinded visual assessments of all TxWBS planar and SPECT/CT images (form available upon request).

**TxWBS semi-quantitative assessment**

Blinded semi-quantitative analysis of I-131 uptake was performed using OsiriX™ (version 8.5 Lite; Pixmeo, Geneva, Switzerland) on both planar and SPECT/CT images. On anterior planar images a region-of-interest (ROI) was drawn around the thyroid region, carefully excluding any physiological uptake or pixels outside patient body contours. Counts within the ROIs were measured. Next, counts were measured within a pre-set, fixed-size rectangular ROI in three background locations: shoulder, thigh and liver, being comparable to the thickness of the neck and/or distant from high iodine uptake regions. Next, mirrored ROIs were copied to the posterior planar image and counts were measured. The following formula was used to compute the geometric mean for iodine uptake on planar imaging per patient:

$$[{geometric mean]}_{region}=\sqrt{\sum[count{s]}_{region,anterior}*[count{s]}_{region,posterior}}$$

On SPECT/CT imaging a 3-dimensional volume-of-interest (VOI) was semi-automatically drawn around the thyroid bed using the iso-contour option in OsiriX™. Any physiological uptake or uptake from distant metastases was manually excluded from the VOI. Next, a predefined fixed-volume spherical background VOI (ø 20 mm) was drawn in the shoulder. Counts within the VOIs were measured. The background measurements were used to correct for inter-patient variability, including intensity of iodine uptake for different I-131 doses and time-specific variances. Together, the semi-quantitative measurements allowed for the analysis of four thyroid-to-background uptake-ratios: thyroid-to-shoulder (planar and SPECT/CT), thyroid-to-thigh (planar) and thyroid-to-liver (planar).

Subsequently, the size of the thyroid remnant was semi-quantitatively assessed for each of the ratios by calculating a background- and area-corrected (area, size in mm^2^, or volume in mm^3^ for SPECT/CT) value for the relative additional iodine uptake in the thyroid bed (thy) above background (b), further referred to as remnant-to-background ratio (TRB ratio), using the following formula:

$TRB ratio = \frac{{[counts]}_{\mathrm{thy}}-\frac{[count{s]}_{b}}{{[area]}_{b}}*{[area]}_{thy}}{\frac{[count{s]}_{b}}{{[area]}_{b}}*{[area]}_{thy}} = \frac{[count{s]}_{thy}}{[count{s]}_{b}}*\frac{[{area]}_{b}}{{[area]}_{thy}}-1$

As such, a higher TRB ratio represents higher uptake and an TRB ratio of 0 represents no iodine uptake in the remnant above the background uptake.

**Location of the ROI’s and VOI’s**

- Planar shoulder: superior border of the ROI defined at one sixth of the length of the arm, perpendicular to and starting from the base of the neck till the middle of the wrist joint, drawn in the arm which is contralateral to the highes t uptake in the neck.
- Planar liver: centre of the ROI at one fourth from the lateral side of the maximal width of the liver and one third from the cranial side of the maximal length of the liver.
- Planar thigh: centre of the ROI at two thirds from the cranial side of the length of the thigh, perpendicular and starting from the bladder till the cranial border of the knee joint.
- SPECT/CT shoulder: a muscle component of the contralateral shoulder, five centimetres below the head of the humerus (approximately at the location of the infraspinatus and deltoid muscle.

**Fig A. Example of placement of ROIs (left) and VOIs (right).**


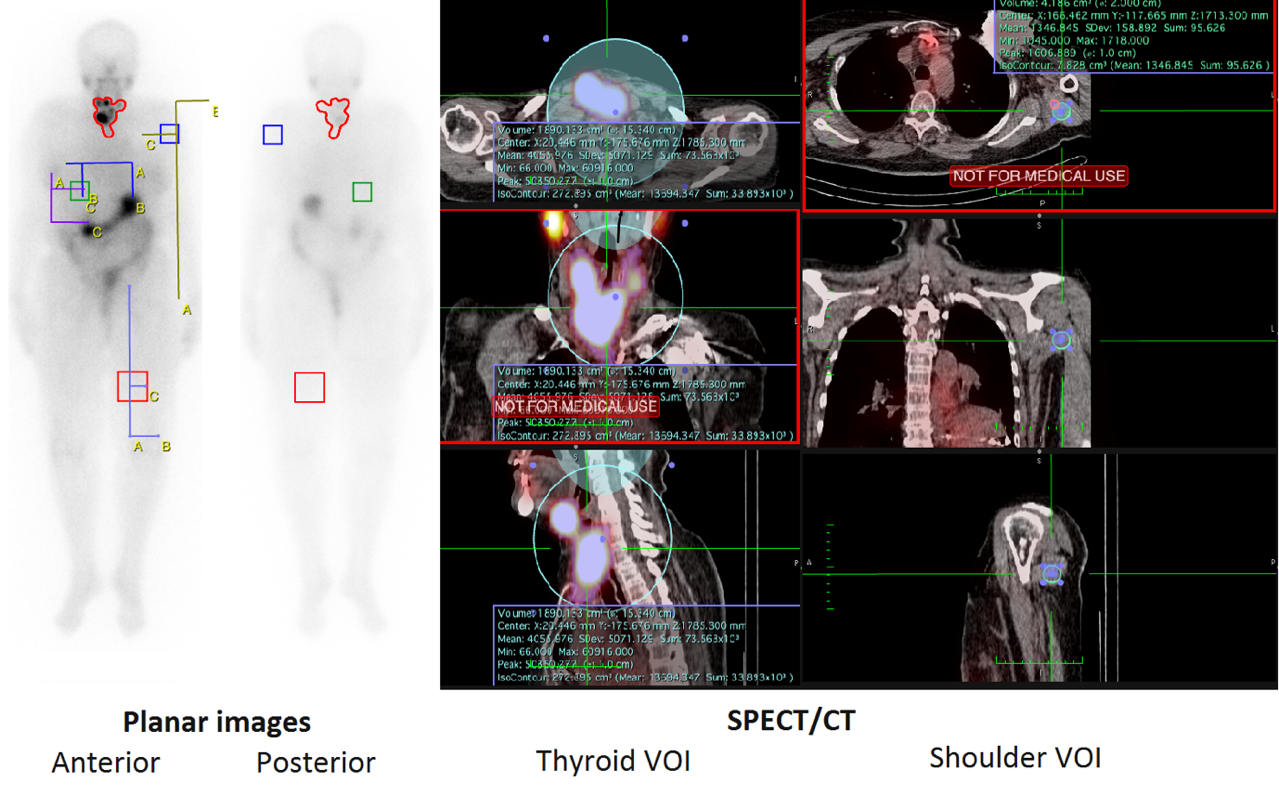


**Semi-quantitative thyroid remnant size**

All TRB ratios except the thyroid-to-liver ratio were intercorrelated: SPECT/CT shoulder vs. planar shoulder: R²=0.471, p=0.000; SPECT/CT vs. planar thigh R²=0.386, p=0.000; planar shoulder vs. planar thigh: R²=0.925, p=0.000 (Fig B).

ROC-curve analysis was performed to determine a cut-off TRB ratio and corresponding sensitivity and specificity for thyroid remnant size to predict ablation success. The SPECT/CT TRB ratio had the highest sensitivity and specificity to predict successful ablation, as demonstrated by a 63% area under the curve (Fig C). As literature also notes SPECT/CT as superior to planar images because of its attenuation correction and ability to correlate iodine avid foci to CT structures, the SPECT/CT based TRB ratio was used in all subsequent analyses [4,5]. SPECT/CT data were available in 87 of 97 patients. The median TRB ratio, representing the size of the thyroid remnant, was 11.6 (IQR: 7.07-28.7).

**
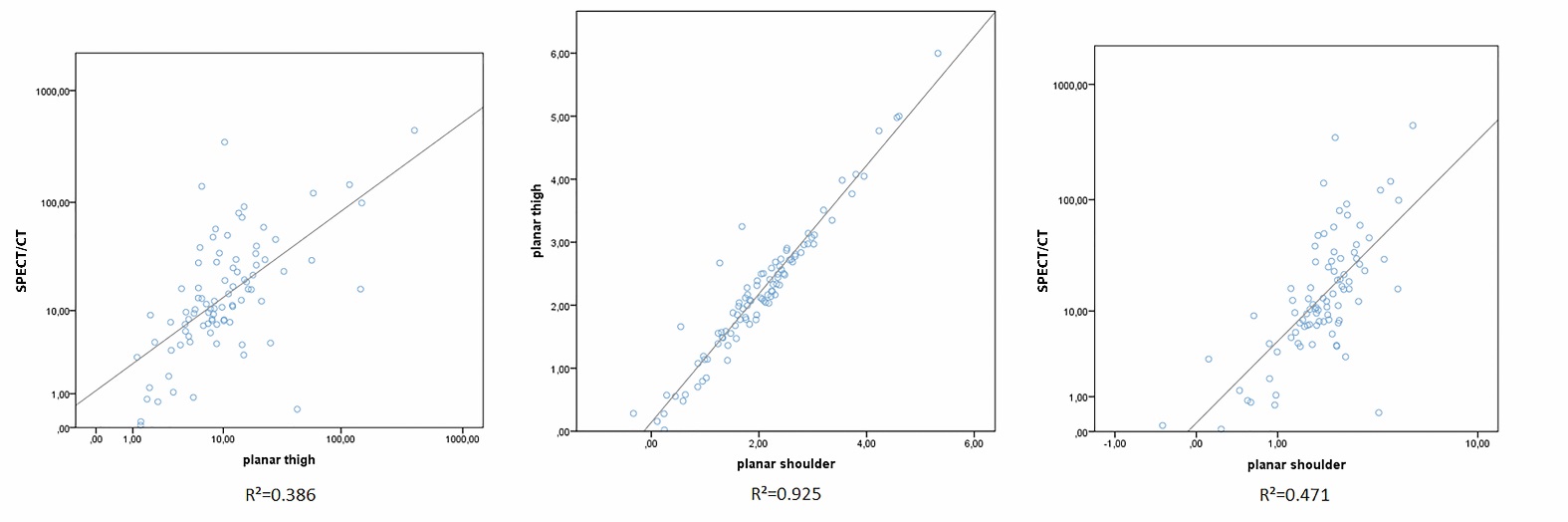
**

**Fig B. Intercorrelation of various TRB ratio methods.** Comparative scatterplots for intercorrelation of various TRB ratio methods to assess the semi-quantitative size of the thyroid remnant

**
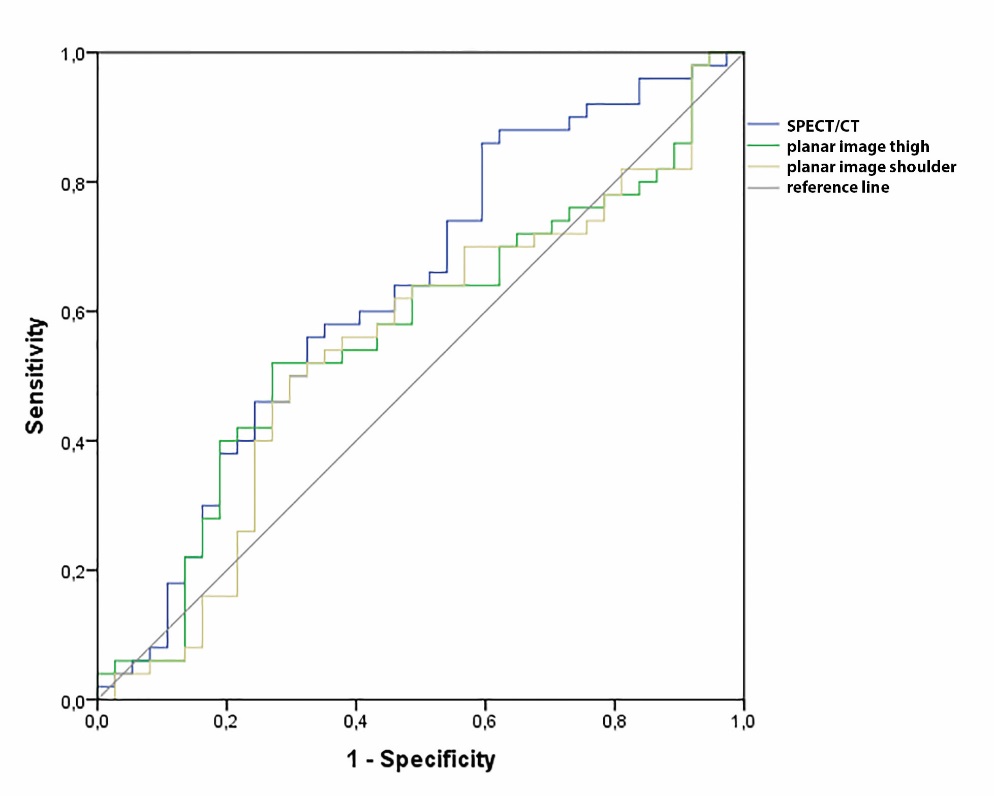
**

**Fig C. ROC curve analysis of TRB ratio methods.** ROC-curve demonstrating the sensitivity and specificity of each of the TRB ratios to predict treatment success

**References**

1. Spencer, C.A. Clinical review: Clinical utility of thyroglobulin antibody (TgAb) measurements for patients with differentiated thyroid cancers (DTC). *J Clin Endocrinol Metab* **2011**, *96*, 3615-3627, doi:10.1210/jc.2011-1740.

2. Haugen, B.R.; Alexander, E.K.; Bible, K.C.; Doherty, G.M.; Mandel, S.J.; Nikiforov, Y.E.; Pacini, F.; Randolph, G.W.; Sawka, A.M.; Schlumberger, M., et al. 2015 American Thyroid Association Management Guidelines for Adult Patients with Thyroid Nodules and Differentiated Thyroid Cancer: The American Thyroid Association Guidelines Task Force on Thyroid Nodules and Differentiated Thyroid Cancer. *Thyroid* **2016**, *26*, 1-133, doi:10.1089/thy.2015.0020.

3. Spencer, C.A. Assay of Thyroid Hormones and Related Substances. In *Endotext*, De Groot, L.J., Chrousos, G., Dungan, K., Feingold, K.R., Grossman, A., Hershman, J.M., Koch, C., Korbonits, M., McLachlan, R., New, M., et al., Eds. South Dartmouth (MA), 2000.

4. Tharp, K.; Israel, O.; Hausmann, J.; Bettman, L.; Martin, W.H.; Daitzchman, M.; Sandler, M.P.; Delbeke, D. Impact of 131I-SPECT/CT images obtained with an integrated system in the follow-up of patients with thyroid carcinoma. *Eur J Nucl Med Mol Imaging* **2004**, *31*, 1435-1442, doi:10.1007/s00259-004-1565-2.

5. Ruf, J.; Lehmkuhl, L.; Bertram, H.; Sandrock, D.; Amthauer, H.; Humplik, B.; Ludwig Munz, D.; Felix, R. Impact of SPECT and integrated low-dose CT after radioiodine therapy on the management of patients with thyroid carcinoma. *Nucl Med Commun* **2004**, *25*, 1177-1182.
