# Supplementary material for: Radioiodine in Differentiated Thyroid Carcinoma: Do We Need Diagnostic Pre-Ablation Iodine-123 Scintigraphy to Optimize Treatment?
Source: Diagnostics (Basel). 2021 Mar 19;11(3):553. doi: 10.3390/diagnostics11030553 (PMC8003652; doi:10.3390/diagnostics11030553)
Supplement: Supplementary file 1 [file diagnostics-11-00553-s001.zip › diagnostics-1148010-supplementary (1)/Table S1.docx]

**S1 Table. Individual patient data.** Individual patient data concerning treatment and findings on TxWBS

|  | **Histopathology** | **Tumor size (mm)** | **protocol**  **recommended**  **I-131 dose (GBq)** | **administered I-131 dose (GBq)** | **Findings on TxWBS** | **Clinically relevant findings?** | **Recommended management change*** | **Outcome at 9 months** |
| --- | --- | --- | --- | --- | --- | --- | --- | --- |
| 1 | pT1aN0 multifocal cPTC, R0 | 7 | 3.7 | 3.7 | small remnant; 1 level 6 lymph node <10mm | yes | higher I-131 dose (5.55 GBq) | Successful |
| 2 | pT1aNx multifocal cPTC, R1 | 4 | 5.55 | 5.55 | large remnant | yes | consider additional surgery or two-step I-131 ablation | Successful |
| 3 | pT1aN1a multifocal cPTC (largest tumour 8mm), R0, 1 lymph nodes+ | 41 | 3.7 | 5.55 | large remnant | yes | consider additional surgery or two-step I-131 ablation, lower cumulative dose (3.7 GBq) | Successful |
| 4 | pT1aN1a multifocal cPTC, R0, 8 lymph nodes+ | 10 | 3.7 | 5.55 | none | no | lower I-131 dose (3.7 GBq) | Successful |
| 5 | pT1aN1b cPTC, R0, 4 lymph nodes+ with extranodal growth | 6 | 5.55 | 5.55 | large remnant | yes | consider additional surgery or two-step I-131 ablation | Successful |
| 6 | pT1aN1b multifocal cPTC, R0, 10 lymph nodes+ | 4 | 5.55 | 5.55 | large remnant | yes | consider additional surgery or two-step I-131 ablation | Unsuccessful |
| 7 | pT1aN1b multifocal cPTC, R0, 2 lymph nodes+ with extranodal growth | 6 | 5.55 | 5.55 | remnant | no | no adjustments to management | Unsuccessful |
| 8 | pT1aN1b multifocal cPTC, R0, 3 lymph nodes+ | 2 | 5.55 | 5.55 | remnant | no | no adjustments to management | Unsuccessful |
| 9 | pT1bN0 FVPTC, R0 | 18 | 1.1 | 3.7 | remnant | no | lower I-131 dose (1.1 GBq) | Successful |
| 10 | pT1bN0 FVPTC, R0 | 11 | 1.1 | 3.7 | none | no | lower I-131 dose (1.1 GBq) | Unsuccessful |
| 11 | pT1bN0 FVPTC, R1 | 20 | 5.55 | 3.7 | remnant | no | higher I-131 dose (5.55 GBq) | Unsuccessful |
| 12 | pT1bN0 FTC-OV, R0 | 15 | 3.7 | 3.7 | remnant; 1 level 6 lymph node <10mm | yes | higher I-131 dose (5.55 GBq) | Successful |
| 13 | pT1bN0 multifocal cPTC, R0 | 16 | 3.7 | 3.7 | large remnant | yes | consider additional surgery or two-step I-131 ablation | Unsuccessful |
| 14 | pT1bN0 multifocal PTC, R1 | 13 | 5.55 | 3.7 | remnant; 1 level 6 lymph node <10mm | yes | higher I-131 dose (5.55 GBq) | Unsuccessful |
| 15 | pT1bN0 multifocal diffuse sclerosing PTC, R1 | 11 | 5.55 | 5.55 | remnant; 3 level 3/6 lymph nodes <10mm | yes | higher I-131 dose (7.4 GBq) | Unsuccessful |
| 16 | pT1bNx cPTC, R0 | 10 | 1.1 | 3.7 | remnant | no | lower I-131 dose (1.1 GBq) | Successful |
| 17 | pT1bNx FVPTC, R0 | 16 | 1.1 | 1.1 | large remnant | yes | consider additional surgery or two-step I-131 ablation, higher cumulative dose (3.7 GBq) | Successful |
| 18 | pT1bNx FVPTC, R0 | 12 | 1.1 | 3.7 | remnant | no | lower I-131 dose (1.1 GBq) | Successful |
| 19 | pT1bNx multifocal diffuse sclerosing PTC, R1 | 11 | 5.55 | 3.7 | remnant | no | higher I-131 dose (5.55 GBq) | Unsuccessful |
| 20 | pT1bN1a cPTC, R0, 1 lymph nodes+ | 19 | 1.1 | 5.55 | none | no | lower I-131 dose (1.1 GBq) | Unsuccessful |
| 21 | pT1bN1a multifocal cPTC, R0, 4 lymph nodes+ | 13 | 3.7 | 3.7 | remnant | no | no adjustments to management | Unsuccessful |
| 22 | pT1bN1b cPTC, R0, 16 lymph nodes+ with extranodal growth | 18 | 5.55 | 5.55 | large remnant | yes | consider additional surgery or two-step I-131 ablation | Successful |
| 23 | pT1bN1b cPTC, R0, 8 lymph nodes+ | 20 | 5.55 | 3.7 | remnant; 1 level 6 lymph node <10mm | yes | higher I-131 dose (5.55 GBq) | Unsuccessful |
| 24 | pT1bN1b cPTC, R1, 8 lymph nodes+ with extranodal growth | 18 | 5.55 | 5.55 | remnant | no | no adjustments to management | Successful |
| 25 | pT1bN1b multifocal cPTC, R0, 4 lymph nodes+ | 20 | 5.55 | 5.55 | remnant | no | no adjustments to management | Successful |
| 26 | pT1bN1b multifocal cPTC, R1, 5 lymph nodes+ with extranodal growth | 20 | 5.55 | 7.4 | large remnant | yes | consider additional surgery or two-step I-131 ablation, lower cumulative dose (5.55 GBq) | Unsuccessful |
| 27 | pT1N1b cPTC, R1, 5 lymph nodes+ | 3 | 5.55 | 5.55 | small remnant;  1 level 2a lymph nodes <10mm | no | no adjustments to management | Unsuccessful |
| 28 | pT2N0 cPTC, R0 | 26 | 1.1 | 1.1 | large remnant | yes | consider additional surgery or two-step I-131 ablation, higher dose (3.7 GBq) | Successful |
| 29 | pT2N0 cPTC, R0 | 28 | 1.1 | 3.7 | large remnant | yes | consider additional surgery or two-step I-131 ablation | Successful |
| 30 | pT2N0 cPTC, R0 | 35 | 1.1 | 3.7 | remnant | no | lower I-131 dose (1.1 GBq) | Unsuccessful |
| 31 | pT2N0 FTC, R0 | 35 | 1.1 | 3.7 | remnant; 1 level 6 lymph node <10mm | yes | higher I-131 dose (5.55 GBq) | Successful |
| 32 | pT2N0 FTC, R0 | 21 | 1.1 | 3.7 | large remnant | yes | consider additional surgery or two-step I-131 ablation | Unsuccessful |
| 33 | pT2N0 FTC, R0 | 38 | 1.1 | 5.55 | remnant; distant  metastasis | yes | higher I-131 dose (7.4 GBq) | Unsuccessful |
| 34 | pT2N0 FVPTC, R0 | 25 | 1.1 | 3.7 | remnant | no | lower I-131 dose (1.1 GBq) | Unsuccessful |
| 35 | pT2N0 FTC-OV, R0 | 25 | 3.7 | 5.55 | remnant | no | lower I-131 dose (3.7 GBq) | Successful |
| 36 | pT2N0 multifocal cPTC, R0 | 30 | 3.7 | 3.7 | remnant | no | no adjustments to management | Successful |
| 37 | pT2N0 multifocal cPTC, R0 | 29 | 3.7 | 3.7 | none | no | no adjustments to management | Successful |
| 38 | pT2N0 multifocal cPTC, R0 | 35 | 3.7 | 3.7 | none | no | no adjustments to management | Unsuccessful |
| 39 | pT2N0 multifocal FTC, R0 | 38 | 3.7 | 3.7 | none | no | no adjustments to management | Successful |
| 40 | pT2N0 multifocal FVPTC, R0 | 24 | 3.7 | 1.1 | remnant; 1 level 2a lymph node <10mm | yes | higher I-131 dose (5.55 GBq) | Unsuccessful |
| 41 | pT2N0 multifocal FVPTC, R0 | 21 | 3.7 | 3.7 | large remnant; 1 level 7 lymph node >10mm | yes | consider additional surgery or two-step I-131 ablation, consider lymph nodes surgery, higher cumulative dose (7.4 GBq) | Unsuccessful |
| 42 | pT2Nx cPTC, R0 | 40 | 1.1 | 3.7 | large remnant | yes | consider additional surgery or two-step I-131 ablation | Successful |
| 43 | pT2Nx FTC, R0 | 30 | 1.1 | 3.7 | remnant; 1 level 4 lymph node <10mm | yes | higher I-131 dose (5.55 GBq) | Successful |
| 44 | pT2Nx FTC, R1 | 20 | 1.1 | 5.55 | large remnant; 1 level 3 lymph node <10mm | yes | consider additional surgery or two-step I-131 ablation | Unsuccessful |
| 45 | pT2Nx FVPTC, R0 | 22 | 1.1 | 1.1 | large remnant | yes | consider additional surgery or two-step I-131 ablation, higher cumulative dose (3.7 GBq) | Successful |
| 46 | pT2Nx multifocal cribriform variant PTC, R0 | 40 | 3.7 | 3.7 | remnant; 1 level 6 lymph node <10mm | yes | higher I-131 dose (5.55 GBq) | Successful |
| 47 | pT2N1b cPTC, R1, 4 lymph nodes+ | 25 | 5.55 | 5.55 | large remnant | yes | consider additional surgery or two-step I-131 ablation | Successful |
| 48 | pT2N1b multifocal cPTC, R0, 14 lymph nodes+ | 35 | 5.55 | 5.55 | remnant | no | no adjustments to management | Unsuccessful |
| 49 | pT2N1b multifocal cPTC, R0, 29 lymph nodes+ | 30 | 5.55 | 5.55 | remnant | no | no adjustments to management | Successful |
| 50 | pT2N1b multifocal cPTC, R1, 3 lymph nodes+ | 29 | 5.55 | 5.55 | remnant; 3 level 3/4/6 lymph nodes | yes | higher I-131 dose (7.4 GBq) | Unsuccessful |
| 51 | pT3N0 cPTC, R0 | 55 | 3.7 | 3.7 | large remnant | yes | consider additional surgery or two-step I-131 ablation | Successful |
| 52 | pT3N0 cPTC, R1 | 25 | 5.55 | 3.7 | remnant; 1 level 4 lymph node <10mm | yes | higher I-131 dose (5.55 GBq) | Unsuccessful |
| 53 | pT3N0 diffuse sclerosing PTC, R0 | 11 | 3.7 | 3.7 | remnant | no | no adjustments to management | Unsuccessful |
| 54 | pT3N0 FTC, R0 | 60 | 3.7 | 3.7 | remnant | no | no adjustments to management | Successful |
| 55 | pT3N0 FTC, R0 | 69 | 3.7 | 3.7 | remnant, distant  metastasis | yes | higher I-131 dose (7.4 GBq) | Unsuccessful |
| 56 | pT3N0 FTC, R0 | 42 | 3.7 | 3.7 | remnant; 1 level 6 lymph node <10mm | yes | higher I-131 dose (5.55 GBq) | Successful |
| 57 | pT3N0 FTC, R1 | 45 | 5.55 | 5.55 | large remnant | yes | consider additional surgery or two-step I-131 ablation | Unsuccessful |
| 58 | pT3N0 FTC, R1 | 30 | 5.55 | 5.55 | large remnant | yes | consider additional surgery or two-step I-131 ablation | Successful |
| 59 | pT3N0 FVPTC, R0 | 53 | 3.7 | 5.55 | large remnant | yes | additional surgery or two-step I-131 ablation, lower cumulative dose (3.7 GBq) | Unsuccessful |
| 60 | pT3N0 FTC-OV, R0 | 35 | 3.7 | 3.7 | large remnant | yes | consider additional surgery or two-step I-131 ablation | Unsuccessful |
| 61 | pT3N0 multifocal cPTC, R0 | 17 | 3.7 | 3.7 | large remnant | yes | consider additional surgery or two-step I-131 ablation | Successful |
| 62 | pT3N0 multifocal cPTC, R1 | 28 | 5.55 | 5.55 | remnant | no | no adjustments to management | Successful |
| 63 | pT3N0 multifocal FVPTC, R0 | 46 | 3.7 | 3.7 | remnant | no | no adjustments to management | Successful |
| 64 | pT3N0 poorly differentiated PTC, R0 | 50 | 3.7 | 7.4 | remnant | no | lower I-131 dose (3.7 GBq) | Unsuccessful |
| 65 | pT3N0 poorly differentiated PTC, R1 | 11 | 3.7 | 5.55 | large remnant | yes | consider additional surgery or two-step I-131 ablation, lower cumulative dose (3.7 GBq) | Unsuccessful |
| 66 | pT3Nx FTC, R1 | 50 | 5.55 | 5.55 | large remnant | yes | consider additional surgery or two-step I-131 ablation | Unsuccessful |
| 67 | pT3Nx multifocal cPTC, R0 | 55 | 3.7 | 5.55 | remnant | no | lower I-131 dose (3.7 GBq) | Unsuccessful |
| 68 | pT3Nx multifocal FVPTC, R1 | 17 | 5.55 | 3.7 | large remnant | yes | consider additional surgery or two-step I-131 ablation, higher cumulative dose (5.55 GBq) | Successful |
| 69 | pT3Nx multifocal tall cell variant PTC, R1 | 12 | 5.55 | 5.55 | remnant | no | no adjustments to management | Successful |
| 70 | pT3N1 cPTC, R0, 2 lymph nodes+ of which 1 in situ | 42 | 7.4 | 7.4 | remnant; 1 level 4 lymph node <10mm | no | no adjustments to management | Unsuccessful |
| 71 | pT3N1a cPTC, R0, 1 lymph nodes+ | 18 | 3.7 | 5.55 | remnant | no | lower I-131 dose (3.7 GBq) | Successful |
| 72 | pT3N1a cPTC, R0, 2 lymph nodes+ | 27 | 3.7 | 5.55 | remnant | no | lower I-131 dose (3.7 GBq) | Successful |
| 73 | pT3N1a diffuse sclerosing PTC, R0, 2 lymph nodes+ | 45 | 3.7 | 5.55 | large remnant; 1 level 7 lymph node <10mm | yes | consider additional surgery or two-step I-131 ablation | Unsuccessful |
| 74 | pT3N1a multifocal cPTC, R1, 1 lymph nodes+ | 42 | 5.55 | 5.55 | remnant; 1 level 7 lymph node <10mm | no | no adjustments to management | Unsuccessful |
| 75 | pT3N1a multifocal FVPTC, R0, 10 lymph nodes+ with extranodal growth | 15 | 3.7 | 5.55 | remnant | no | lower I-131 dose (3.7 GBq) | Unsuccessful |
| 76 | pT3N1a oncocytic PTC, R1, 3 lymph nodes+ | 30 | 5.55 | 3.7 | remnant; 1 level 5b lymph node <10mm;  distant metastasis | yes | higher I-131 dose (7.4 GBq) | Unsuccessful |
| 77 | pT3N1b cPTC, R0, 1 lymph nodes+ | 35 | 5.55 | 5.55 | remnant | no | no adjustments to management | Unsuccessful |
| 78 | pT3N1b cPTC, R0, 10 lymph nodes+ with extranodal growth | 14 | 5.55 | 5.55 | remnant | no | no adjustments to management | Successful |
| 79 | pT3N1b cPTC, R0, 4 lymph nodes+ with extranodal growth | 110 | 5.55 | 5.55 | remnant | no | no adjustments to management | Unsuccessful |
| 80 | pT3N1b multifocal cPTC, R1, 4 lymph nodes+ with extranodal growth | 11 | 5.55 | 5.55 | remnant | no | no adjustments to management | Successful |
| 81 | pT3N1b cPTC, R1, 16 lymph nodes+ with extranodal growth | 20 | 5.55 | 5.55 | remnant | no | no adjustments to management | Unsuccessful |
| 82 | pT3N1b cPTC, R1, 2 lymph nodes+ | 23 | 5.55 | 5.55 | remnant | no | no adjustments to management | Unsuccessful |
| 83 | pT3N1b cPTC, R1, 2 lymph nodes+ with extranodal growth | 28 | 5.55 | 5.55 | remnant | no | no adjustments to management | Unsuccessful |
| 84 | pT3N1b cPTC, R1, 2 lymph nodes+ with extranodal growth | 14 | 5.55 | 5.55 | remnant | no | no adjustments to management | Unsuccessful |
| 85 | pT3N1b cPTC, R1, unknown number of lymph nodes+ | 4 | 5.55 | 5.55 | remnant; 1 level 7 lymph node >10mm | yes | consider lymph nodes surgery or higher I-131 dose (7.4 GBq) | Unsuccessful |
| 86 | pT3N1b diffuse sclerosing PTC, R1, 16 lymph nodes+ with extranodal growth | 90 | 5.55 | 5.55 | remnant; 3 level 2b/4/7 lymph nodes <10mm; distant metastasis | yes | higher I-131 dose (7.4 GBq) | Unsuccessful |
| 87 | pT3N1b multifocal cPTC, R1, 10 lymph nodes+ with extranodal growth | 9 | 5.55 | 5.55 | remnant | no | no adjustments to management | Unsuccessful |
| 88 | pT3N1b multifocal cPTC, R1, 15 lymph nodes+ | 10 | 5.55 | 5.55 | remnant | no | no adjustments to management | Unsuccessful |
| 89 | pT3N1b multifocal cPTC, R1, 25 lymph nodes+ with extranodal growth | 33 | 5.55 | 5.55 | remnant; 1 level 2a lymph node <10mm, distant metastasis | yes | higher I-131 dose (7.4 GBq) | Unsuccessful |
| 90 | pT3N1b multifocal cPTC, R1, 4 lymph nodes+ | 12 | 5.55 | 5.55 | remnant; 2 level 2b/3 lymph nodes <10mm | yes | higher I-131 dose (7.4 GBq) | Unsuccessful |
| 91 | pT3N1b multifocal diffuse sclerosing PTC, R1, 1 lymph nodes+ | 16 | 5.55 | 5.55 | remnant | no | no adjustments to management | Unsuccessful |
| 92 | pT3N1b multifocal FVPTC, R1, 7 lymph nodes+ with extranodal growth | 10 | 5.55 | 5.55 | large remnant | yes | consider additional surgery or two-step I-131 ablation | Successful |
| 93 | pT4aN0 FVPTC, R1 | 42 | 5.55 | 7.4 | remnant, distant  metastasis | no | no adjustments to management | Unsuccessful |
| 94 | pT4aNx tall cell variant PTC, R1 | 65 | 5.55 | 7.4 | remnant; 3 level 3+6 lymph nodes, two >10mm | yes | consider lymph nodes surgery | Unsuccessful |
| 95 | pT4aN1a multifocal poorly differentiated PTC, R1, 1 lymph nodes+ | 21 | 5.55 | 5.55 | remnant | no | no adjustments to management | Unsuccessful |
| 96 | pT4aN1b classic PTC, R0, 9 lymph nodes+ | 38 | 5.55 | 7.4 | remnant; 1 level 7 lymph node <10mm | no | lower I-131 dose (5.55 GBq) | Unsuccessful |
| 97 | pT4aN1b FTC, R0, 16 lymph nodes+ | 70 | 5.55 | 5.55 | remnant; 3 level 3/6 lymph nodes, two >10mm | yes | consider lymph nodes surgery or higher I-131 dose (7.4 GBq) | Successful |

*: in accordance with Dutch national guidelines, had TxWBS findings been known before RTA administration. cPTC: classic type papillary thyroid carcinoma. FTC: follicular thyroid carcinoma. FTC-OV: follicular thyroid carcinoma, oncocytic variant. FVPTC: follicular variant papillary thyroid carcinoma. Lymph nodes+: tumour-positive lymph node. R0: radical resection. R1: irradical resection.
